# Observational study pelvic ultrasound a useful tool in the diagnosis and differentiation of precocious puberty in Chinese girls

**DOI:** 10.1097/MD.0000000000010092

**Published:** 2018-03-09

**Authors:** Xiaoduo Wen, Denggui Wen, Hui Zhang, Huifeng Zhang, Yi Yang

**Affiliations:** a Department of Ultrasound in Obstetrics and Gynecology; b Cancer Institute, the Fourth Hospital of Hebei Medical University; c Department of Pediatrics Endocrinology, the Second Hospital of Hebei Medical University, Shi Jiazhuang, Hebei, China.

**Keywords:** central precocious puberty, cervix, Chinese girls, isolated precocious puberty, ovaries, pelvic ultrasound, uterine

## Abstract

Rapid and noninvasive diagnosis on and differentiation between normal, central precocious puberty (CPP), and isolated precocious puberty (IPP) is imperative before a decision can be made with gonadotropin-releasing hormone (GnRH) agonist treatment. Our study aims to evaluate such a role by pelvic ultrasound.

We consecutively enrolled 84 cases of IPP (59 with premature thelarche/ pubarche and 25 with premature menarche), 47 CPP, and 177 age-matched normal controls. The IPP and CPP were diagnosed by clinical examination and GnRH-stimulation test and confirmed by over 2 years’ follow-up. All subjects underwent pelvic ultrasound examination for length, width, thickness, volume of uterine/cervix/ovaries, fundal/cervical thickness ratio, endometrial thickness, and averaged maximal diameter of largest follicles. Statistical comparisons of these sonographic parameters between disease groups were made according to age intervals.

It was found that between CPP and normal girls, 10 and 12 ultrasound parameters differed significantly in the >6 to 8 and >8 to 10 years age interval, respectively. Cervical thickness and endometrial thickness was the best discriminating parameter in the 2 intervals by receiver operating characteristic (ROC) curve analysis, and the cutoff, sensitivity and specificity associated with was 0.73 cm, 93.30%, 85.70%, and 0.26 cm, 76.92%, 100%, respectively. Between CPP and IPP, 2 and 5 parameters differed significantly in the >6 to 8 and >8 to 10 years age interval. Cervical length was the best discriminating parameter in both age intervals. The cutoff, sensitivity, and specificity associated were 1.49 cm, 93.33%, 55.17%, and 1.88 cm, 100%, 71.43%, respectively; Finally between normal and IPP girls, 4, 7, and 5 parameters differed significantly in the 0 to 6, >6 to 8, and >8 to 10 years intervals, respectively. Ovarian thickness, ovarian width, and cervix thickness was the best parameter for the 3 age interval respectively, and the cutoff, sensitivity and specificity associated were 0.98 cm, 76.46%, 84.85%, 1.39 cm, 85.71%,73.81%, and 0.75 cm, 90.48%, 64.21%, respectively.

Our results indicate that pelvic ultrasonography could serve as a complementary tool for differentiation between normal girls and girls with different forms of sexual precocity in China. The best discriminating parameter changes according to precocity forms and age intervals.

## 1 Introduction

Precocious puberty in girls is clinically manifested by the development of secondary sexual characteristics before the age of 8 years. Due to increasing environmental endocrine interfering agents in food and food package, the number of patients referred to pediatric endocrinology clinics for the evaluation of early pubertal signs is rising in recent years in China.^[1]^ The disease is heterogeneous; it may take the form of central precocious puberty (CPP), premature thelarge, premature puberche, or premature menarche. The latter 3 forms are isolated precocious puberty (IPP). CPP is associated with premature activation of the hypothalamic gonadotropin-releasing hormone (GnRH) pulse generator.^[2]^ It can lead to a compromised final adult height because of accelerated bone maturation.^[3]^ Therefore timely treatment with GnRH agonist to suppress the hypothalamic–pituitary–ovarian axis is important.^[4]^ IPPs on the other hand are not associated with early epiphyseal closure and do not require therapy, but may mimic the early symptoms of CPPs and cause diagnostic difficulties.^[5]^

Although GnRH-stimulation test is considered the ‘gold standard’ for differentiating IPP from CPP, and despite its high specificity, its sensitivity is relatively low, because occasionally IPP will progress into CPP in later times.^[6,7]^ Moreover, there is disagreement as to the criteria for interpretation.^[8]^ Considering pelvic ultrasound examination of the internal genitalia is rapid and non-invasive, it is important to explore its role in diagnosis and differentiation of various precocity forms. Although similar studies have been performed in western countries,^[9–13]^ a few are reported about Chinese girls. This study aims to investigate the distribution of pelvic ultrasound parameters among Chinese girls and to assess the diagnostic and discriminating ability about precocity, and to establish reliable cutoff limits.

## 2 Patients and methods

### 2.1 Cases and controls

Our study invited girls visiting the Pediatric Endocrine Outpatient Clinic of the Second Hospital of Hebei Medical University between January 2008 and December 2010 for examination of signs of precocious puberty, which include appearance or history of appearance of breast development and/or pubic hair before 8 years, or menarche before 10 years. The study was approved by the ethics committee of Hebei Medical University. A written informed parental consent was obtained in all cases. All the girls were physically assessed with regard to their breast and pubic hair development according to Tanner staging^[14]^ by HF Zhang. Briefly, Tanner staging for breast is, stage I: no glandular tissue, areola follows the skin contours of the chest (prepubertal); stage II: breast bud forms, with small area of surrounding glandular tissue, areola begins to widen; stage III: breast begins to become more elevated, and extends beyond the borders of the areola, which continues to widen but remains in contour with surrounding breast; stage IV: increased breast size and elevation, areola and papilla form a secondary mound projecting from the contour of the surrounding breast; stage V: breast reaches final adult size, areola returns to contour of the surrounding breast, with a projecting central papilla. For pubic hair, stage I: no pubic hair at all (prepubertal); stage II: small amount of long, downy hair with slight pigmentation on the labia majora (females); stage III: hair becomes more coarse and curly, and begins to extend laterally (11.5–13); stage IV: adult–like hair quality, extending across pubis but sparing medial thighs; stage V: hair extends to medial surface of the thighs. Height measurements were made with the use of a commercial stadiometer and height velocity, evaluated on a minimum of a 6-month period, was calculated. Bone age was assessed by radiographs of the left hand and wrist as described by Greulich and Pyle.^[15]^ Basal levels of luteinizing hormone (LH), follicle-stimulating hormone (FSH), and estradiol, and GnRH-stimulated LH and FSH levels were evaluated using commercial radio-immuno assays. The GnRH-stimulation test was performed by injecting GnRH at a dose of 2.5 μg/kg intravenously before measuring LH and FSH 30 and 60 minutes later. The result of the test was considered ‘pubertal’ if the ratio of peak LH versus peak FSH was ≥0.6 or peak LH was ≥3.3 to 5.0 IU/L.^[16]^

Premature thelarche was diagnosed by the appearance of breast development before 8 years old, bone age within mean chronological age ± 2 standard deviation (SD), prepubertal height velocity <6 cm/year and prepubertal response to the GnRH test. Premature pubarche was diagnosed by the appearance of pubic hair and/or axillary hair before 8 years old, bone age within mean chronological age ± 2 SD, prepubertal height velocity <6 cm/year and prepubertal response to the GnRH test. Premature menarche was diagnosed by the appearance of menarche before 10 years old, bone age within mean chronological age ± 2 SD, prepubertal height velocity <6 cm/year and prepubertal response to the GnRH test. Lastly, CPP was diagnosed by the appearance of breast buds before 8 years old, and accompanied by 1 or more in below: accelerated bone age (≥mean chronological age + 2 SD), accelerated height velocity (≥6 cm/year), pubic and/or axillary hair or menses, and was confirmed by pubertal response to GnRH testing. Magnetic resonance imaging of the hypophyseal– hypothalamic area was done in every so diagnosed CPP patient to exclude central nervous system abnormality. All patients diagnosed with isolated premature pubarche accepted adrenocorticotropic hormone stimulation test to exclude congenital adrenal hyperplasia.

Transabdominal pelvic ultrasound scans were performed by XW and HZ in all girls before the commencement of any kind of hormonal therapy. A Philips P700 ultrasound set (Philips Medical Systems Inc., Bothell, WA) equipped with a 5-MHz convex-array broad-band transducer and a 7.5-MHz linear-array small parts transducer was used. Drinking water was prescribed to all girls to produce a full bladder to serve as an acoustic window through which the pelvic internal genitalia were examined. Parameters of the uterine, cervix, and ovaries include length, width, thickness, volume (ellipsoid volume: V (cm^3^) = length (cm) × width (cm) × thickness (cm) × 0.5236), and fundal/cervical thickness ratio, endometrial thickness, and averaged maximal diameter of the largest follicle.

One hundred and thirty-one girls with different forms of precocious puberty (59 with premature thelarche/ pubarche, 25 with premature menarche, and 47 with CPP) were enrolled in the study. All cases were followed-up for at least 2-years to confirm the diagnosis. Girls with non-idiopathic sexual precocity were excluded from the study. Additionally, 177 normal prepubertal girls aged 0 to 10 years were recruited from primary schools in Shijiazhuang city. This control group were examined concurrently by the same physician and ultrasound doctors, and served as a control group. The age range (minimum-maximum) for CPP, IPP, and controls was 1.7 to 9.9, 2.8 to 9.7, and 4.2 to 10.0 years, respectively. To control the cofounding effect of age, comparison of ultrasound measurements between CPP, IPP, and normal girls were restricted within 0 to 6, >6 to 8, and >8 to 10 years intervals (Table 1).

**Table 1:**
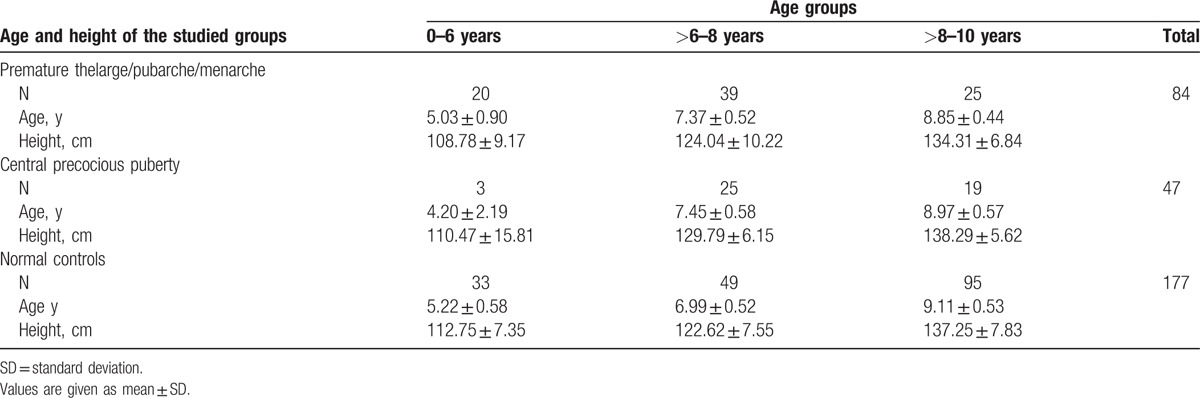
Age and height of the studied groups.

### 2.2 Statistics

Statistical analysis and graphic presentation were performed using the statistics package for social studies (SPSS) for Windows software (IBM SPSS Software V20.0 statistical package). Mann-Whitney *U*-test was used to test the difference in means between 2 groups for continuous variables, if the variance had been tested to be equal. Otherwise Tamhane's T_2_ test was employed. A *P*-value less than.05 were considered statistically significant. To determine optimal cutoff points for discriminating between various forms of sexual precocity, receiver operating characteristics (ROC) curves analysis were used.

## 3 Results

### 3.1 Groups and demographics

On ultrasound, a uterus or a cervix was identified in 98.70% (304/308) and 88.96% (274/308), and both ovaries were visualized in 90.91% (208/308) of the girls. The maximum diameter of the largest follicle was measurable in both ovaries in 74.03% (228/308). Endometrial echo was visualized in 37.33% (115/308), and the endometrial thickness was measured in 33.12% (102/308). Measurability of the internal genitalia increases with increasing age. In the 0 to 6 years age interval, only 3 CPP cases were available, and cervix and endometrial echo was identified in only 1 case, therefore evaluation of the role of pelvic examination in this age interval was impossible. In the >6 to 8 years and >8 to 10 years age intervals, all sonographic parameters were evaluated.

As measurements of length, width, thickness, and volumes of the right and the left ovary did not differ significantly in individual subjects [eg, the mean disparity ± SD for ovarian volume was 0.315 ± 2.02 cm^3^; 95% confidence interval (CI) was −0.514 to +0.681], the average value of both ovaries (right ovary + left ovary)/2 was calculated and used in analysis.

Because all comparison of sonographic parameters between various groups were made in an age-restricted manner, premature thelarge, premature pubarche, and premature menarche cases were combined into 1 IPP group in the final analysis to overcome sample size scarcity.

There was no significant difference in height between either 2 among IPP, CPP, and normal controls, in any age intervals, except in the >6 to 8 years age interval, in which CPP cases were significantly higher than either IPP or the normal controls (*P* < .01) (Table 1).

The pelvic ultrasonographic measurements of the length, width, thickness, and volume of uterine/cervix/ovary all increase as the chronological age increases, but the increase is slow and small until the age of 8 years old. After that, all the parameters increase significantly. As a result, none of the pelvic sonographic parameters was significantly different between 0-6 years and >6 to 8 years, but all parameters in the >8 to 10 years interval were significantly higher than either the 0 to 6 years or >6 to 8 years old (Table 2).

**Table 2:**
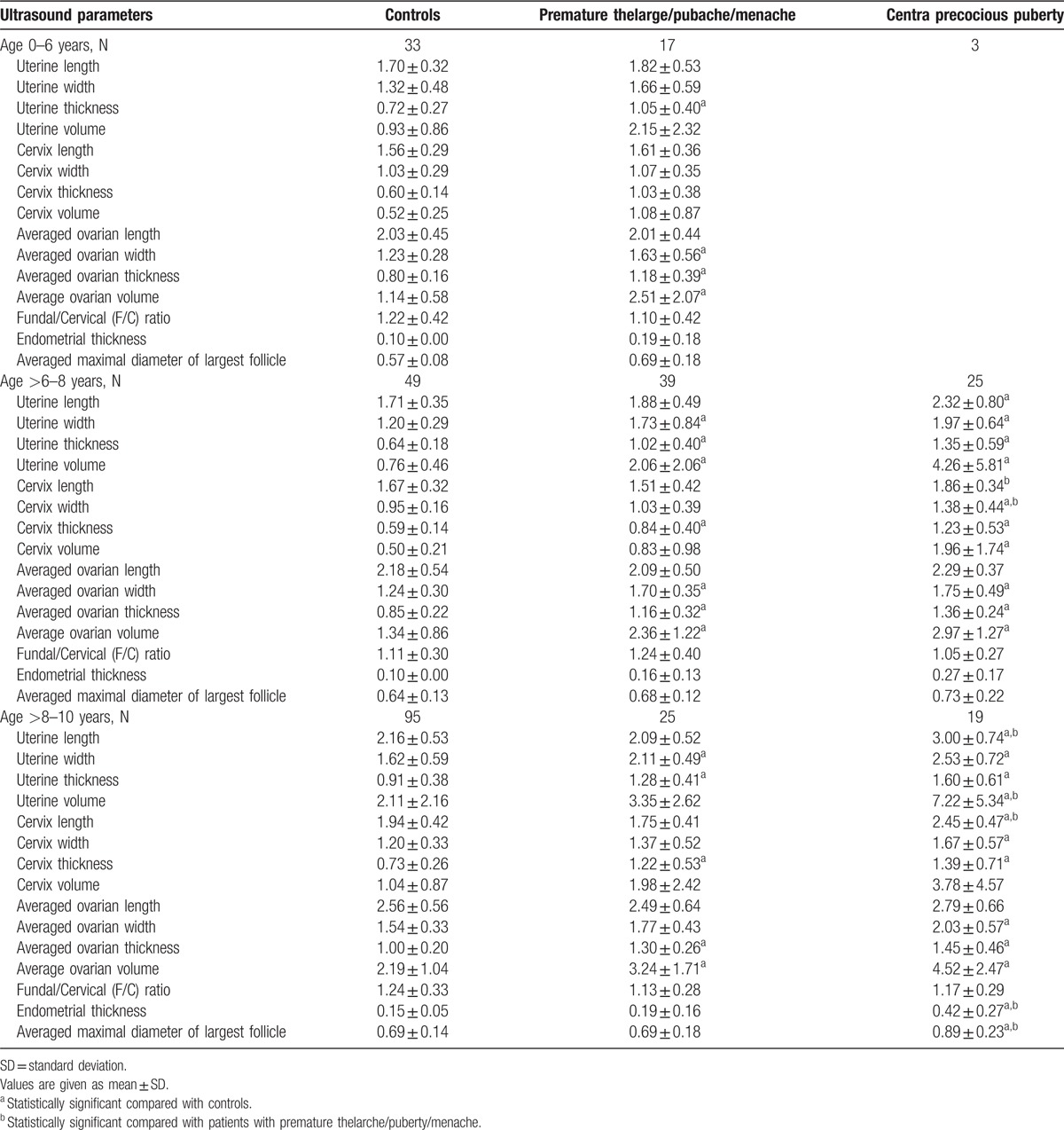
Comparison of length, width, thickness, and volume of the uterine, uterine cervix, ovary, and thickness of the endometrium between patient with central precocious puberty, premature thelarge/pubache/menarche, and normal controls by the age groups of 0–6 years, ≥6–8 years, and ≥8–10 years.

### 3.2 Significant parameters found between CPP, IPP, and normal girls and the best discriminating parameter according to ROC curve analysis

Of the totally 15 ultrasound parameters, 10 and 12 were significantly elevated in CPP cases as compared to normal controls in the >6 to 8 or the >8 to 10 age interval, respectively (Table 2). These parameters were potentially valuable for differentiating between CPP and normal controls. The number of significantly different variables between IPP and normal controls in the 0 to 6, >6 to 8, and >8 to 10 years age intervals was 4, 7, and 5, respectively (Table 2).

To diagnose CPP from normal controls in the >6 to 8 years age interval, significant variables consist of ovarian width, thickness, volume, cervical width, thickness, volume, and uterine length, width, thickness, volume (Table 2). Among these 10 significant variables, the best variable was cervix thickness, as judged by the largest value of area under the ROC curve (0.958); a cutoff of 0.73 cm had a sensitivity of 93.30% and a specificity of 85.70% (Table 3). In the >8 to 10 years age interval, in addition to the 10 significant measurements listed above for the >6 to 8 years age interval (with the exception of cervical volume which was replaced by cervical length), endometrial thickness and the averaged maximal diameter of largest follicle were also significant, as a results of growing measurability with increasing age (Table 2). Endometrial thickness was the best parameter with the largest value of area under the ROC curve (0.933). A cutoff of 0.26 cm had a sensitivity of 76.92% and a specificity of 100% (Table 3).

**Table 3:**
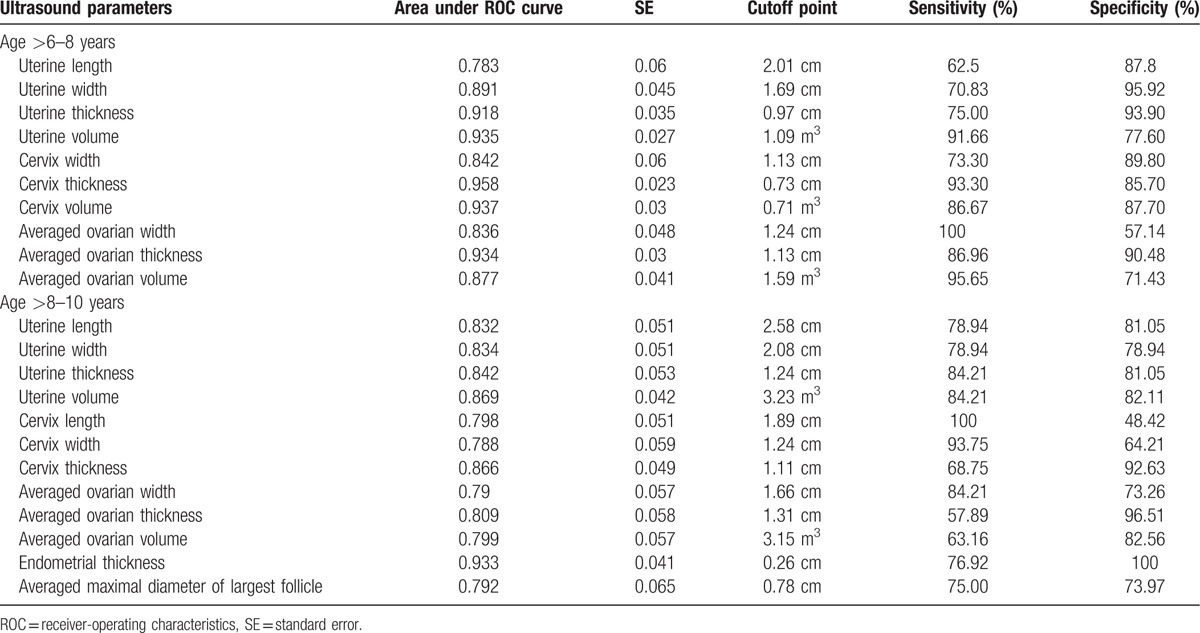
ROC curve parameters and cutoff point value for identifying central precocious puberty patients from normal girls by pelvic ultrasound examination variables.

Compared to normal girls, patients with IPP had significantly elevated ovarian width, thickness, volume, and uterine thickness in the 0 to 6 year age interval (Table 2). Ovary thickness carried the largest area under the ROC curve (0.806), a cutoff of 0.98 cm had a sensitivity of 76.46% and a specificity of 84.85% (Table 4); in the age group of >6 to 8 years, ovarian width, thickness, volume, uterine width, thickness, volume, and cervix thickness were significant variables (Table 2). Ovarian width had the largest area under the ROC curve (0.843), a cutoff of 1.39 cm carried a sensitivity of 85.71% and a specificity of 73.81% (Table 4). Finally in the >8 to 10 years group, ovarian thickness, volume, uterine width, thickness, and cervical thickness were significant parameters (Table 2). Cervix thickness had the largest area under the ROC curve (0.841), and was the best parameter. A cutoff of 0.745 cm had a sensitivity of 90.48% and a specificity of 64.21% (Table 4).

**Table 4:**
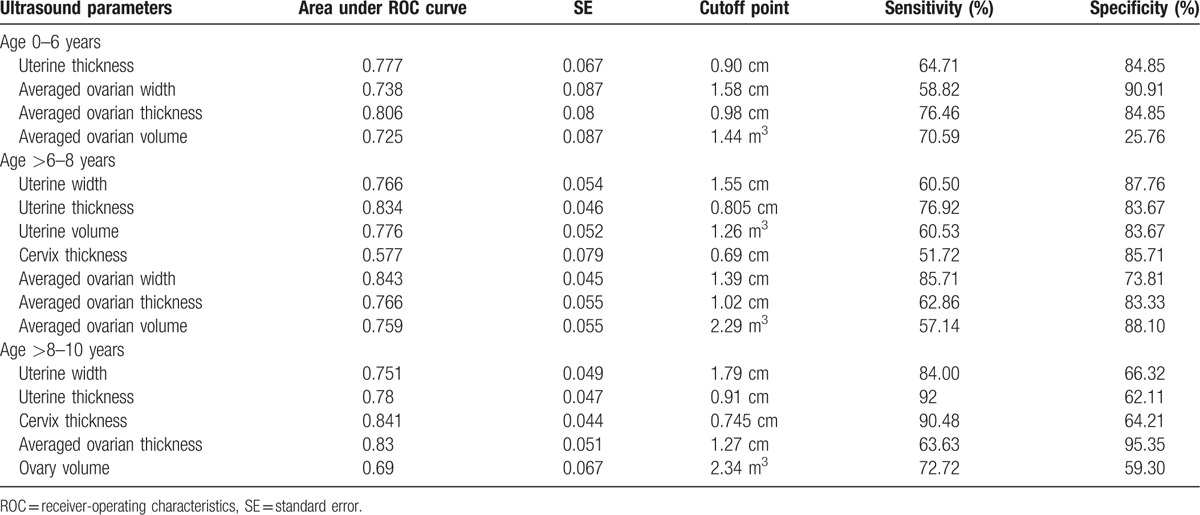
ROC curve parameters and cutoff point value for identifying premature thelarge/pubache/menache cases from normal girls by pelvic ultrasound examination variables.

Between CPP and IPP cases, cervical length and width were significant discriminating parameters in the>6 to 8 age interval (Table 2). A cutoff of 1.49 cm for cervical length, which had a relatively larger value of area under the ROC curve of 0.764, had a sensitivity of 93.33% and a specificity of 55.17% (Table 5). In the >8 to 10 years interval, uterine length, volume, cervical length, endometrial thickness, and the averaged maximal diameter of largest follicle were significant parameters (Table 2). The best discriminating variable was again cervical length, with the largest value of area under the ROC curve (0.893), and a cutoff of 1.88 cm carried a sensitivity of 100% and a specificity of 71.43% (Table 5).

**Table 5:**
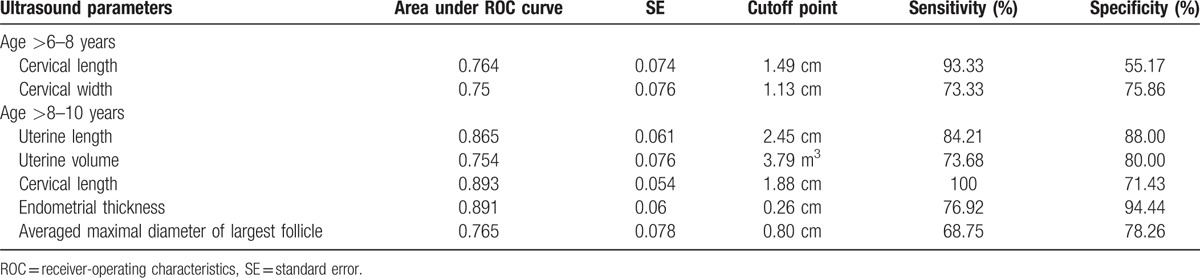
ROC curve parameters and cutoff point value for identifying central precocious puberty patients from premature thelarge/pubache/menache cases by pelvic ultrasound examination variables.

### 3.3 Parameters with specific discriminating ability

**Figure 1:**
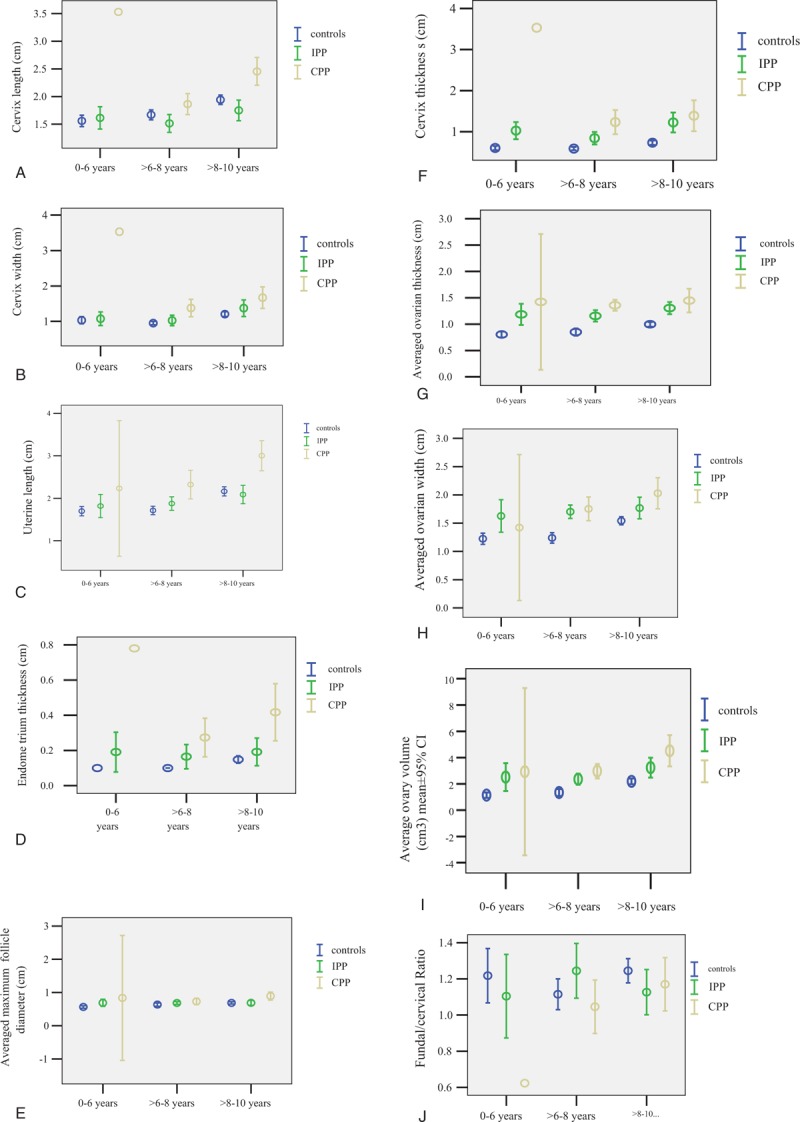
A-J. Comparisons of mean ± 95% confidence interval (CI) of representative pelvic ultrasound examination variables between normal girls, isolated precocious puberty (IPP), and central precocious puberty (CPP) cases in 3 age groups.

**Figure 2:**
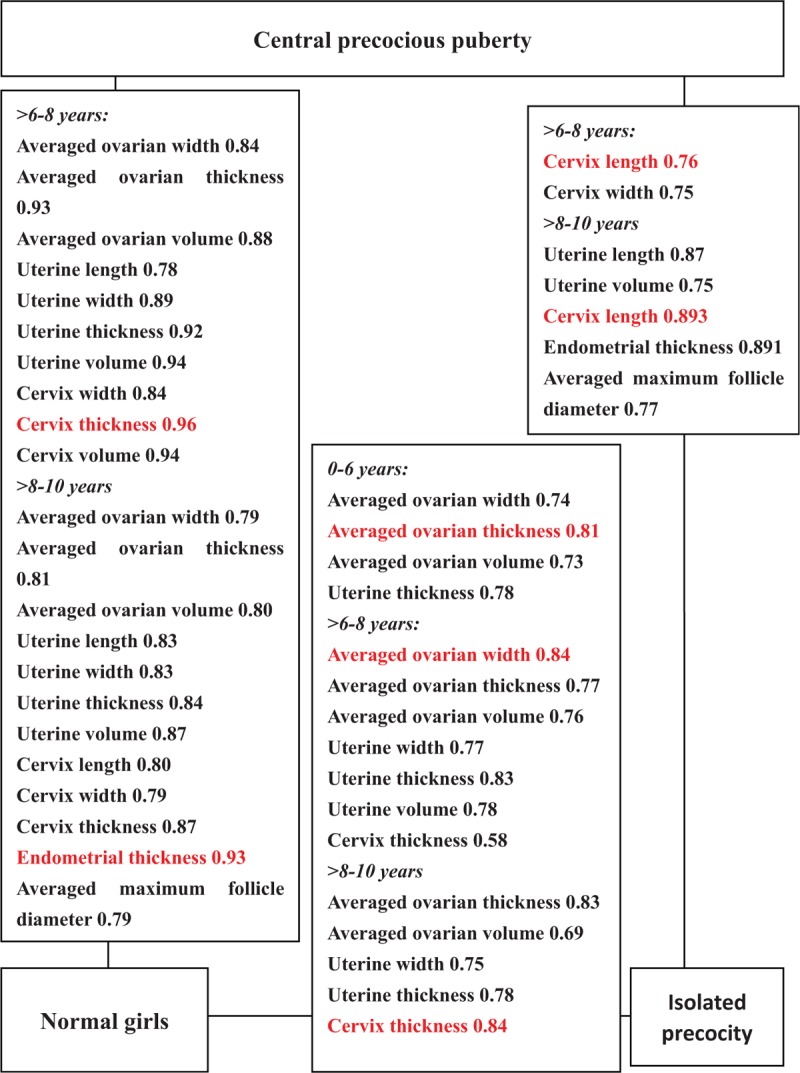
Significant ultrasonographic parameters and the value of area under receiver operating characteristic curve for differentiating between central precocious puberty (CPP), isolated precocious puberty (IPP), and normal control according to age intervals.

Although significant differences in ultrasound measurements are commonly observed between CPP, IPP, and normal controls, some are specific. For instance, though measurements of the ovary are significantly higher in either IPP or CPP cases as compared to normal controls, these parameters are not significantly higher in CPP than in IPP, and do not discriminate between them [Figs. 1 (G–I) and 2]. As the exact opposite, cervix length, width, uterine length, endometrial thickness, and the averaged maximal diameter of largest follicle are significantly higher in CPP than in both IPP and normal controls, but these parameters are not significantly higher in IPP than in normal controls. Because of the significant difference found between CPP and IPP, these variables may be used to extinguish between them [Figs. 1 (A–E) and 2].

Several sonographic parameters differ significantly between IPP and CPP suggests they are sonographically different (Table 2, Fig. 2). As reflected in the results of ROC analyses, the best discriminating parameter between IPP and normal controls in the 0 to 6, >6 to 8, and >8 to 10 years age interval was ovarian thickness, width, and cervical thickness, respectively (Table 4), but that between CPP and normal control in the >6 to 8 and >8 to 10 years age interval was cervical thickness and endometrial thickness (Table 3). Since IPP can be occasionally regarded as an early stage of CPP, it may be postulated that ovarian measurements are more sensitive to estrogen stimulation and may have increased significantly at IPP stage, and remained at the high level thereafter, whereas cervical thickness or endometrial thickness may have increased significantly only when IPP progresses into CPP. This assumption is supported by the fact that although both CPP and IPP exhibit statistically significantly elevated ultrasound parameters than normal controls, the significantly elevated parameters are different. Figure 2 depicts these significantly increased parameters: from between IPP and normal control to between CPP and normal control, as the age interval increases, more and more parameters of internal genitalia become significantly different: firstly between IPP and normal, in the 0 to 6 years age interval, significantly difference was observed only with ovarian parameters (width, thickness, volume) and uterine thickness, but in the >6 to 8 age interval, uterine width, volume, cervical thickness were added as significant parameters; secondly between CPP and normal control, significant differences were found in 10 measurements of ovaries/uterine/cervix in the >6 to 8 years age interval, and in the >8 to 10 years age interval, endometrial thickness and the averaged maximal diameter of largest follicle also became significantly different. All these seem to suggest enlargement of internal genitalia occurs gradually in the order of ovary, uterine and cervix in CPP development. In IPP cases, ovarian width, thickness, volume, uterine wide, thickness, volume, and cervical thickness may be significantly elevated, but cervical length, width, uterine length, endometrial thickness, and the averaged maximal diameter of largest follicle are not significantly higher yet, these second group of parameters become significantly higher only with CPP cases, and these parameters are significantly different between CPP and IPP cases.

The variables of endometrial thickness and the averaged maximal diameter of largest follicle are measurable only in the >8 to 10 age interval and they are significantly different between CPP and control or between CPP and IPP, but not between IPP and normal controls. Therefore they are candidate parameters for discrimination between CPP and normal or between CPP and IPP only in the >8 to 10 age interval [Figs. 1 (D, E) and 2].

In summary of the value of separate best parameters as shown in red color in Figure 2, cervix thickness was the best parameter for discriminating CPP from controls in the >6 to 8 years age interval, a cutoff of 0.73 cm had a sensitivity of 93.30% and a specificity of 85.70% (Table 3). In the >8 to 10 year interval, it was the second most efficient parameter (next only to endometrial thickness), a cutoff of 1.11 cm had a sensitivity of 68.75% and a specificity of 92.63% (Table 3). In addition, cervix thickness was also the most efficient parameter to identify IPP from normal controls in the >8 to 10 years age interval, a cutoff of 0.75 cm had a sensitivity of 90.48% and a specificity of 64.21% (Table 4).

Endometrial thickness was the best parameter for discriminating CPP from normal girls and the second best parameter for discriminating CPP from IPP, both in the 8 to 10 years interval. In the first case, a cutoff of 0.26 cm had a sensitivity of 76.92% and specificity of 100% (Table 3), and in the second case, a cutoff of 0.26 cm had a sensitivity of 76.92% and a specificity of 94.44% (Table 5).

Cervix length was the best parameter for discriminating CPP from IPP cases in both >6 to 8 years and >8 to 10 years age intervals, the cutoff, sensitivity and specificity was 1.49 cm, 93.33%, 55.17% and 1.88 cm, 100%, 71.43%, respectively (Table 5).

The averaged ovarian thickness and width was the best parameter for differentiating between IPP and normal controls in the 0 to 6, >6 to 8 years interval respectively (Table 4). But for the differentiation between CPP and controls, ovarian measurements are inferior to cervix thickness in the >6 to 8 years interval, or are inferior to endometrial thickness in the >8 to 10 years interval. Because no ovarian measurement was significant different between CPP and IPP cases, these parameters could not be used to differentiate between CPP and IPP cases.

## 4 Discussion

Criteria for the diagnosis of CPP by GnRH stimulation test have been non-consistent; an LH peak ≥25 IU/L or an LH peak/FSH peak ≥1 is considered to be a pubertal response to GnRH stimulation by Palmert et al,^[17]^ whereas the criteria set by the Chinese Ministry of Health, possibly to cover ethnic variation, was an LH peak ≥3.3 to 5.0 IU/L or an LH peak/FSH peak ≥0.6.^[16]^ In present study, we used the relatively looser criteria by the Chinese Ministry of Health to reduce the rate of false negativity, and we followed IPP cases for at least 2 years to reclassify the 4 cases who had showed a prepubertal response to the GnRH stimulation test at diagnosis but progressed to CPP during follow-up.

Because ultrasonographic measurements of internal genitalia increase with age, to facilitate comparisons, we analyzed the difference in ultrasound parameters between CPP, IPP, and controls according to age range, and calculated cutoff limits for each subgroup separately.

Previous studies in western countries often found ovary volume having the highest discriminating power for the identification of CPP patients.^[11–13,18–20]^ In present study of Chinese girls, ovary volume was one of the significant parameters in discriminating between CPP and control, but was not the best parameter. Instead we found cervical thickness, volume, uterine volume were the most 3 efficient parameters for the >6 to 8 years, and endometrial thickness, uterine volume, and cervical thickness were the 3 most powerful parameters for the >8 to 10 years old (Table 3). These results suggest the best sonographic parameter and its ‘optimal cutoff value may change according to populations. However, a similar study with a larger sample size performed in Israel reported a similar finding as us. They found that uterine volume is one of the most efficient parameter for both predicting CPP and distinguishing between CPP and premature thelarche.^[10]^

Early studies reported fundal/cervical ratio is significantly higher in CPP than in normal controls and is a significant discriminating parameter,^[10,18]^ but in our study, because CPP cases had both significantly increased fundal and cervical thickness than the normal controls, and the increase in cervical thickness is even larger, therefore the CPP cases had a somehow identical or even lower fundal/cervical ratio than the controls (Figure 1, J).

Badouraki et al^[18]^ reported uterine length was the best parameter for distinguishing between CPP and premature thelarche cases, a cutoff of 3.19 cm and of 3.83 cm gave sensitivities of 85.7 and 82.4% and specificities of 91.7 and 90.9% for the 0 to 6 and *>*6 to 8 years age intervals, respectively. In our study, uterine length is the third most efficient parameter for distinguishing between CPP and IPP for the >8 to 10 years age interval, a cutoff of 2.45 cm gave a sensitivity of 84.21 and specificity of 88.00%. Instead of uterine length, we found cervix length is the best parameter for discriminating between CPP and IPP in both >6 to 8 and >8 to 10 years age intervals, a cutoff of 1.49 cm had a sensitivity of 93.33% and a specificity of 55.17%, and a cutoff of 1.88 cm carried a sensitivity of 100% and a specificity of 71.43%, respectively (Table 5). In addition to cervix length, cervix width was also a useful parameter in the >6 to 8 years age interval, and uterine length, volume, endometrial thickness, and averaged maximal diameter of largest follicle were significant parameters in the >8 to 10 years age interval (Table 5). Because early discrimination between CPP and IPP is imperative before GnRH agonist treatment, and satisfactory treatment depends upon early differentiation, these new differentiating sonographic parameters found in Chinese girls deserve to be further explored.

In present study, we performed pelvic sonographic examination at the time of clinical examinations and gonadotropin-releasing hormone-stimulation test by which the diagnosis of IPP or CPP was established. At this moment, measurements of the internal genitalia actually provide cross-sectional information on ovary, uterine, cervix associated with IPP and CPP in the natural history of CPP. Comparison of such sonographic data provides a glimpse of sex organs development in the course of CPP. In this way, we found ovarian measurements are significantly higher in IPP than in normal controls, but are not significantly higher in CPP than in IPP cases. As the exact opposite, cervical length, width, uterine length, endometrial thickness, and average maximum diameter of largest follicle are significantly elevated only in CPP cases. These parameters are significantly higher in CPP than in IPP cases, and can be utilized to distinguish between CPP and IPP cases. From these, we postulate that ovarian parameters are sensitive to hormonal stimulation and increase significantly at the early (isolated) stage, whereas cervical length, width, uterine length, endometrial thickness, and average maximum diameter of largest follicle do not increase significantly until at the CPP stage.

Our study is limited by a relative small sample size. There were only 47 CPP cases participated in the study. The number of CPP patient at age range 0 to 6 years (N = 3) is too small for any meaningful analysis. Another limitation may be related to the retrospective nature of study design. The significant differences in ultrasonographic parameters found between CPP, IPP, and normal controls may be caused by some confounding factors than precocious puberty. Therefore the diagnostic value is limited. However, this does not prevent ultrasound from being considered as a complimentary noninvasive method.

## 5 Conclusions

In conclusion, our results suggest ultrasound pelvic examination could serve as a complementary tool for the differentiation of CPP, IPP, and normal Chinese girls, but the efficacy changes according to disease stage and age intervals. Our study is the first to note the significant discriminating power associated with cervical parameters in Chinese girls, but need to be confirmed by others.

## References

[1] Li XD Yan X Chen LH Evaluations of puberty sexual development in girls by intraluminal ultrasound. Natl Med J China 2012;92:1187–9. 22883007

[2] Lee PA Central precocious puberty. An overview of diagnosis, treatment, and outcome. Endocrinol Metab Clin North Am 1999;28:901–18. 10609126 10.1016/s0889-8529(05)70108-0

[3] Adan L Chemaitilly W Trivin C Factors predicting adult height in girls with idiopathic central precocious puberty: implications for treatment. Clin Endocrinol (Oxf) 2002;56:297–302. 11940040 10.1046/j.1365-2265.2002.01488.x

[4] Ritz’en EM Early puberty: what is normal and when is treatment indicated? Horm Res 2003;60 (Suppl 3):31–4. 14671393 10.1159/000074497

[5] Stanhope R Premature thelarche: clinical follow-up and indication for treatment. J Pediatr Endocrinol Metab 2000;13 (Suppl 1):827–30. 10969928 10.1515/jpem.2000.13.s1.827

[6] Iughetti L Predieri B Ferrari M Diagnosis of central precocious puberty: endocrine assessment. J Pediatr Endocrinol Metab 2000;13 (Suppl 1):709–15. 10969913 10.1515/jpem.2000.13.s1.709

[7] Pescovitz OH Hench KD Barnes KM Premature thelarche and central precocious puberty: the relationship between clinical presentation and the gonadotropin response to luteinizing hormone-releasing hormone. J Clin Endocrinol Metab 1988;67:474–9. 3137242 10.1210/jcem-67-3-474

[8] Neely EK Wilson DM Lee PA Spontaneous serum gonadotropin concentrations in the evaluation of precocious puberty. J Pediatr 1995;127:47–52. 7608810 10.1016/s0022-3476(95)70255-5

[9] Battaglia C Mancini F Regnani G Pelvic ultrasound and color Doppler findings in different isosexual precocities. Ultrasound Obstet Gynecol 2003;22:277–83. 12942501 10.1002/uog.154

[10] de Vries L Horev G Schwartz M Ultrasonographic and clinical parameters for early differentiation between precocious puberty and premature thelarche. Eur J Endocrinol 2006;154:891–8. 16728550 10.1530/eje.1.02151

[11] Griffin IJ Cole TJ Duncan KA Pelvic ultrasound measurements in normal girls. Acta Pediatr 1995;84:536–43. 10.1111/j.1651-2227.1995.tb13689.x 7633150

[12] Haber HP Wollmann HA Ranke MB Pelvic ultrasonography: early differentiation between isolated premature thelarche and central precocious puberty. Eur J Pediatr 1995;154:182–6. 7758513 10.1007/BF01954267

[13] Herter LD Golendziner E Flores JA Ovarian and uterine findings in pelvic sonography: comparison between prepubertal girls, girls with isolated thelarche, and girls with central precocious puberty. J Ultrasound Med 2002;21:1237–46. 12418765 10.7863/jum.2002.21.11.1237

[14] Marshall WA Tanner JM Variations in pattern of pubertal changes in girls. Arch Dis Child 1969;44:291–303. 5785179 10.1136/adc.44.235.291 PMC2020314

[15] Greulich WW Pyle SI Radiographic atlas of skeletal development of the hand and wrist. 2nd edn Stanford, CA: Stanford University Press; 1959.

[16] Chinese Ministry of Public Health. Guideline to the diagnosis and treatment of precocious puberty in China. No 195 by Division of Medical Administration. Chinese J Child Health Care 2011;19:390–2.

[17] Palmert MR Malin HV Boepple PA Unsustained or slowly progressive puberty in young girls: initial presentation and long-term follow-up of 20 untreated patients. J Clin Endocrinol Metab 1999;84:415–23. 10022394 10.1210/jcem.84.2.5430

[18] Badouraki M Christoforidis A Economou I Evaluation of pelvic ultrasonography in the diagnosis and differentiation of various forms of sexual precocity in girls. Ultrasound Obstet Gynecol 2008;32:819–27. 18951545 10.1002/uog.6148

[19] Buzi F Pilotta A Dordoni D Pelvic ultrasonography in normal girls and in girls with pubertal precocity. Acta Pediatr 1998;87:1138–45. 10.1080/080352598750031121 9846915

[20] Stanhope R Adams J Jacobs HS Ovarian ultrasound assessment in normal children, idiopathic precocious puberty, and during low dose pulsatile gonadotrophin releasing hormone treatment of hypogonadotrophic hypogonadism. Arch Dis Child 1985;60:116–9. 3883909 10.1136/adc.60.2.116 PMC1777151

